# The effects of gas flaring as moderated by government quality in leading natural gas flaring economies

**DOI:** 10.1038/s41598-023-38032-w

**Published:** 2023-09-01

**Authors:** Andrew Adewale Alola, Stephen Taiwo Onifade, Cosimo Magazzino, Hephzibah Onyeje Obekpa

**Affiliations:** 1 CREDS-Centre for Research on Digitalization and Sustainability, Inland Norway University of Applied Science, Elverum, Norway; 2 Faculty of Economics, Administrative, and Social Sciences, Nisantasi University, Istanbul, Turkey; 3 Adnan Kassar School of Business, Lebanese American University, Beirut, Lebanon; 4 Department of International Trade and Logistics, Faculty of Economics and Administrative Sciences, KTO Karatay University, Konya, Turkey; 5 School of Finance and Accounting, University of Vaasa, 65200 Vaasa, Finland; 6 Department of Political Science, Roma Tre University, Rome, Italy; 7 Department of Agricultural Economics, Federal University of Agriculture, Makurdi, Nigeria; 8 CIPESS-Center for Innovation in Procurement, Environmental and Social Standards, Federal University of Agriculture, Makurdi, Nigeria

**Keywords:** Climate sciences, Environmental sciences, Energy science and technology

## Abstract

This study seeks to address pertinent economic and environmental issues associated with natural gas flaring, especially for the world's leading natural gas flaring economies (i.e. Russia, Iraq, Iran, the United States, Algeria, Venezuela, and Nigeria). By applying relevant empirical panel and country-specific approaches, the study found that fuel energy export positively impacts economic growth with elasticity of ~ 0.22 to ~ 0.24 for the panel examination. It is further revealed that environmental quality in the panel is hampered by increase in economic growth, gas flaring, fuel energy export, and urbanization. Moreover, for the country-wise inference, government quality desirably moderates economic and environmental aspects of gas flaring in Venezuela and Nigeria, and in Russia and Iran respectively. However, government quality moderates gas flaring to cause economic downturn in the USA. Additionally, economic growth increased with increase in urbanisation (in Iraq and the USA), gas flaring (in Iran and the USA), government quality (only in the USA), and fuel energy export (only in Algeria) while economic growth downturn is due to increase urbanisation in Russia and the USA, increase in fuel energy export in the USA, and increase in government quality in Russia. Meanwhile, environmental quality is worsened through intense carbon dioxide emission from increased urbanisation activity (in Iraq, Iran, Algeria, and Nigeria), increased fuel energy export (in Nigeria), increased natural gas flaring (in Algeria and Nigeria), increased GDP (in Russia, Iran, USA, Algeria, and Venezuela), and high government quality (in Iran). Interestingly, the result revealed that increase in GDP (in Nigeria), increase in urbanisation (in the USA), and increase in gas flaring (in Algeria and Nigeria) dampens environmental quality. Importantly, this study offers policy insight into sustainable approaches in natural gas production, government effectiveness, and regulatory quality.

## Introduction

Humans' increasing reliance on energy sources is not unexpected considering the drive for economic prosperity, population gains, and the competitiveness of the global market structure. Although fossil fuels have long remained a major source of global energy development, the associated environmental drawbacks, and the damning humans' twenty first-century challenge (climate change) have further widened the search for environmentally sustainable energy source(s). While natural gas is a preferred choice of energy input for economic activities compared to oil, neither of the production processes is free from gas flaring and venting, yet natural gas is another significant source of environmental hazard. When natural gas is burned in conjunction with oil production, several pollutants, including carbon dioxide (CO_2_), methane (CH_4_), and black carbon (a major part of particulate matter-PM), are emitted into the environment^1,2^. This practice is known as gas flaring. Although, it is arguable that different factors promote natural gas flaring, in most cases, insufficient infrastructure to locally utilise or transport gases to market, and weak policy regulations against flaring are often cited among other issues^3,4^.

Meanwhile, the release of greenhouse gases (GHG) into the atmosphere constitutes major environmental concerns about gas flaring given the immense evidence of the dangers ahead of humanity if the necessary collective actions are ignored against the backdrop of climate change. Besides, it has also been reported that gas flares “adversely influence human development circumstances” and “damage natural resources and local livelihoods,” as well as “alienate people from their land”^5^. The impact of gas flaring and venting ranges from economic^6,7^ to health and pollution effect^8,9^. Hence, some initiatives have been coming up to create a change in the status quo and ultimately put an end to the gas flaring practices. Notable among these initiatives is the “Zero Routine Flaring by 2030 (ZRF)” by the World Bank which is mainly designed to help halt routine flaring of gases on or before 2030^10^. However, national and sub-national levels decide the rules for gas flaring. As a result, the legality of flaring, the circumstances in which it is permitted, and the requirements for reporting are all quite variable. Thus, many more issues have to be addressed to attain this laudable initiative by the set time of 2030.

Although the recent report implied that approximately 144 billion cubic meters of gas were flared in 2021 (thus suggesting that gas flaring has plateaued in the last 10 years). Ten leading flaring countries reportedly account for 75 percent and 50 percent of global gas flared and oil production, respectively^11^. Specifically, there has been a significant flaring increase in Mexico, Libya, and China recently, while the volume of flared gas has remained relatively at the same levels for world-leading natural gas flaring countries including Russia, Iraq, Iran, the United States, Venezuela, Algeria, and Nigeria. The seven countries investigated in this study jointly account for 65% of global flaring making them significant contributors to the problems caused by gas flaring^12^.

Therefore, this study makes timely contributions to the growing environmental literature by considering the panel of aforementioned world-leading flaring countries in certain ramifications. To begin with, the investigation looks at the economic and environmental impacts of gas flaring from a novel perspective that differs from the previous studies' objectives. Specifically, while considering the role of gas flaring in both economic and environmental sustainability drives in the panel countries, examining the role of government effectiveness and regulation quality (jointly dubbed as government quality) is the central objective of this study. Meanwhile, another objective of the investigation is to examine whether government quality plays a moderating role in affecting the impact of gas flaring on the economic and environmental prosperity of the examined countries. This is important because of the perceived vulnerability of the energy sector to corruption, mismanagement, and inefficiency among the energy exporting countries, especially the developing economies^13–15^.

Moreover, as part of the scope of the investigation, the long-run economic and environmental sustainability aspects of fuel export and urbanization (urban population growth) were also examined. Why the latter factors have been strongly upheld by different empirical studies to influence environmental quality in terms of how they can induce/abate the overall environmental effects of conventional energy consumption^16–19^, hitherto, most of these extant studies have side-lined the need to provide a disaggregated analysis from a gas flaring perspective even though flaring accounts for a sizable portion of annual global carbon emissions and other dangerous atmospheric pollutants^8,20^. Thus, as a major contribution to the literature on gas flaring, the current study draws novelty from the fact that the panel of top global gas flaring countries was likely examined for the first time alongside the examination of the economic and environmental aspects of government quality vis-à-vis government effectiveness and regulatory quality.

The remaining sections are arranged in a specified order in a conventional approach and for easy readership. The related literature is discussed in section “Literature review”, while section “Data, Computation, and Tests” is dedicated to highlighting and describing the dataset and required empirical analysis. In section “Discussion of findings”, the empirical results were discussed, while the concluding section is reserved for summarizing the study and highlighting relevant policy.

## Literature review

Flaring generates waste of energy resources with a significant negative environmental impact and economic losses. A recent^21^ report alarmed on gas flaring and its associated consequences arising from legal, financial, or technical barriers to the growth of the gas market, the inability of the current infrastructure to support its profitable exploitation, or the impossibility of injecting associated gas back into the reservoir. Consequently, severe environmental setbacks have been widely associated with gas flaring arising from energy production^1,2,22^.

As^22^ highlighted, the environmental problems caused by flaring are global, regional, and local. The literature on gas flaring is vast and diverse since it presents studies of an economic, physical, chemical, and environmental nature. In addition, there are various surveys on this topic. Buzcu-Guven and Harriss^23^ reviewed the literature on the flaring and venting of associated natural gas in producing oil fields. Fawole et al.^8^ provided a critical review of the gas flaring-air pollution nexus, focusing on black carbon. Much of the available literature focuses on the environmental impacts; however, Okoye et al.^24^ argue that one of the biggest effects of gas flaring's adverse effects on the environment is the enormous waste of money required to clean up the environment, pay for medical care, and ultimately lose human capital. Ngene et al.^25^ reviewed the role of flaring and venting in the production of oil and gas, while Mansoor and Tahir^26^ reviewed current gas flaring reduction and utilization techniques for the production of energy-efficient fuels. Importantly, Mansoor and Tahir^26^ conclude that an opportunity to be exploited in the use of gas flare is a future clean energy economy, as methane has already shown in the production of energy with the abatement of CO_2_ emissions.

Regarding papers on a single country, several papers inspected the gas flaring in Nigeria, Russia, and Iran, the world's three largest gas-flaring countries. Nwankwo and Ogagarue^27^ analyzed the impact of natural gas flaring on the microclimate and maize yield in the Niger Delta. According to their results, the sand content of the soil, pH, bulk density, and air and soil temperatures increase towards the flare site. Meanwhile, Nwankwo and Ogagarue^28^ estimated a percentage of around 70% as being flared from the produced gas in Nigeria. Anomohanran^6^, analyzing the nexus between GHG emissions and gas flaring in Nigeria between 1999 and 2009, evidenced that the amount of resulting GHG emissions from gas flaring exceeds those due to the consumption of petroleum products, estimating that 11 billion dollars are lost annually to gas flaring in the country^29,30^ analyzed gas flaring programs in Nigeria. Okoro and Okoro^31^ identified crude oil production, gas use investments, and gas flaring regulations as some of the factors responsible for gas flaring-related CO_2_ emissions in the case of Nigeria. The results of^32^ on two Nigerian gas and electricity producing firms evidenced that an annual net profit of 2.68 billion $ can be recovered from reusing gas instead of flaring.

Furthermore, Okoro et al.^7^ investigated the determinants of gas flaring activities in Nigeria over the 1970–2019 period. The time-series results show that gas flaring activities are persistent, while economic growth does not Granger cause gas flaring. Loe and Loe^33^ inspected the outlook for Russian gas flaring using data from 2001 to 2011, concluding that the utilization goal could have been reached in the medium term, crucially depending on political will, given the time of economic insecurity. Anejionu et al.^34^ used a combination of geospatial technologies to estimate how air pollution from gas flaring affects human health and natural ecosystems in the Niger Delta region. The empirical findings show that gas flaring can be considered a significant contributor to air pollution, and the World Health Organization (WHO) limits concentrations that are violated. Moreso, ^35^ highlighted how the evaluation of the data for Iran in the period 2007–2016 suggests that—due to a reduction in oil production and problems with processing equipment—the operational flare recovery projects are operating below their nominal capacity. Additionally, Pourhassan and Taravat^36^ analyzed the nexus among gas flaring volume, oil price, CO_2_ emissions, and the total natural resources for eight developing countries in the 1994–2008 years for eight oil-exporting countries. The study established a positive relationship between oil prices and gas flaring. Elvidge et al.^3^ compared 2015 satellite-derived natural gas flaring data with the national GHG reduction targets, showing that several countries with large volumes of flare gases would be able to meet only partially their nationally determined contributions targets thanks to the reductions. Similarly, Nezhadfard and Khalili-Garakan^37^ indicated that power generation from flare gas could potentially be an effective method for flare gas recovery.

Moreover, the relevance of regulations and their effects on flaring was stressed by^38^, who used an evenemential historical analysis to inspect the politics behind flaring regulations in Texas between 1889 and 2017. The archival research illustrated how the historical-legal developments had created new opportunities for firms to exploit the natural gas extracted legitimately. This result aligns with Nigeria's observed trend from 1970 to 2010, as pinpointed by^39^. Hajilary et al.^40^ highlighted the urgency to operate some profound changes in current public policies regarding the production and processing of oil and gas, also considering the objectives set out in the Kyoto Protocol. ^41^ showed that changes in oil production have led to a strong fluctuation of flaring for over a century. Rodrigues^42^ found focused regulation as the reason for Brazil's mid-term decline in gas flaring from 2009 to 2019 using risk mapping and future predictions. He predicted that with better control, the 100% growth in natural gas output might reduce natural gas flaring to roughly 2% in 2030.

From another perspective^9^, estimated the effect of flaring on human health: this is equivalent to 0.542 h over a lifetime. Dong et al., Dong and Pang^43,44^ remarked on the relevance of several factors such as government regulations, available resources, and local market needs, in the selection of liquefied natural gas (LNG) technology and GTL (gas to liquid). The World Bank supports the “zero routine flaring by 2030” initiative (The World Bank, http://www.worldbank.org/en/programs/zero-routineflaring-by-2030). However, gas flaring regulations are set at national and sub-national levels. In the same vein, Canada’s firm policy of monitoring and regulation was responsible for the 70 percent reduction achieved in 2003. The USA also considers regulatory changes as a means to reduce harmful emissions resulting from gas flaring^45^. In the case of Russia^46^, found that the weakness of both formal institutions and practices within the informal institution exerts a significant impact on gas flaring. Specifically, the result implied that exemptions and non-compliance with regulatory licenses trigger gas flaring by 5%, while unwritten rules, distorting rules, and regulatory gaps (associated with the informal institution) also promote more gas flaring.

### Contribution to the literature

Given the review of this literature above and from the summary of the related cases as depicted in Table 1, it is evident that previous investigations mainly concentrated on oil-exporting countries. Contrary to previously published studies, this study takes a broader perspective by investigating a panel of the seven leading countries on global gas flaring. While the current study narrows the existing gap in the literature by considering the world’s top leading gas flaring countries, the effect and moderating role of government quality vis-à-vis government effectiveness and regulation quality were unearthed for both groups and country-specific cases. Thus, the examined hypotheses in the current context are primarily based on the economic and environmental effects of natural gas flaring, the effect and moderating role of government quality (through government effectiveness and regulation quality).

**Table 1:** Summary of the empirical literature.

Authors	Country	Study period	Empirical strategy
Anejionu et al.^34^	Niger delta region	2000–2013	ADMS
Anomohanran^6^	Nigeria	1999–2009	RA
Motte et al.^9^	47 countries	1994–2016	Calculation of the number of DALYs
Nwanya^28^	Nigeria	1970–2007	GSE
Odjugo and Osemwenkhae^27^	Niger delta region	2005–2006	ANOVA
Okoro et al.^7^	Nigeria	1970–2019	ARDL, BH, ECM, GC
Pourhassan and Taravat^36^	8 oil-exporting countries	1994–2008	Panel FE, RE
Willyard^38^	Texas (USA)	1889–2017	EHA

ADMS, Atmospheric Dispersion Modelling System; ANOVA, Analysis of Variance; ARDL, Auto-Regressive Distributed Lags model; BH, Bayer-Hanck cointegration test; DALYs, Disability-Adjusted Life Years; ECM, Error Correction Model; EHA, Evenemential Historical Analysis; FE, Fixed Effects; GC, Granger Causality tests; GSE, Gross Specific Enthalpy; RA, Reference Approach; RE, Random Effects.

Source: authors’ elaborations.

## Data, computation, and tests

In this study, annual data is collected over the period 2002–2020 for the case of the world's seven countries with the highest volume of natural gas flaring (i.e. Russia, Iraq, Iran, the United States, Algeria, Venezuela, and Nigeria). The dataset employed for the empirical study is the Gross Domestic Product (coded as GDP and measured in international dollars using 2017 purchasing power parity rates), carbon emission (coded as CM and measured in a million tonnes of carbon dioxide), natural gas flaring (coded as GFL and measured in billion cubic metres). In addition, fuel export (coded as FEX and measured as a percentage of merchandise exports), urban population (coded as UB and measured in thousands of people living in urban areas), and government quality, denoted as GQ, are computed by the interaction of two variables, i.e. government effectiveness*regulation quality. Additionally, the interaction of government quality and natural gas flaring (i.e. government quality*natural gas flaring) produced another variable, GQGFL, designed to proxy for government effectiveness in addressing natural gas flaring. While FEX, UB, and GDP were retrieved from the World Development Indicators (WDI) of the^47^, CM and GFL were retrieved from the database of^48^. Meanwhile, the government effectiveness and regulation quality dataset were retrieved from the Worldwide Governance Indicators (WGI) of the Millennium Challenge Corporation of the United States of America^49^.

Before conducting relevant empirical tests, the panel descriptive statistics of the dataset alongside the correlation evidence among the variables, especially between the dependents and explanatory variables, are presented. This information is illustrated in Table 2. From the correlation evidence, it is interesting and desirable to observe that fuel exportation, GDP, and government effectiveness in curbing natural gas flaring have a significant and negative correlation with carbon emissions. On the other hand, urbanisation exhibits a significant and positive correlation with both carbon emissions and GDP.

**Table 2:** Descriptive statistics.

Variable	LnCM	LnGDP	LnGFL	LnFEX	LnUB	LnGQ	LnGQGFL
Mean	5.5863	26.927	2.241	4.0469	17.789	− 0.1449	− 0.441
Median	5.196	26.613	2.265	4.4674	17.729	− 0.0489	− 0.101
Maximum	8.681	30.657	3.173	4.605	19.410	0.588	1.277
Minimum	2.0040	23.811	0.979	0.606	16.655	− 4.653	− 14.215
Std. Dev	1.911	1.658	0.588	0.976	0.855	0.624	1.718
Observations	119	119	119	119	119	119	119
Correlation							
LnCM	1						
*p*-value	–						
LnGDP	− 0.745***	1					
*p*-value	0.000	–					
LnGFL	− 0.097	− 0.028	1				
*p*-value	0.296	0.759	–				
LnFEX	− 0.716***	− 0.857***	0.360***	1			
*p*-value	0.000	0.000	0.000	–			
LnUB	0.608***	0.911***	0.086	− 0.786***	1		
*p*-value	0.000	0.000	0.350	0.000	–		
LnGQ	− 0.124	0.022	− 0.319***	− 0.215**	− 0.118	1	
*p*-value	0.178	0.816	0.000	0.019	0.203	–	
LnGQGFL	− 0.194**	− 0.073	− 0.348***	− 0.117	− 0.190**	0.977***	1
*p*-value	0.034	0.431	0.000	0.206	0.038	0.000	–

Computed by the author. ***, **, and * reflect the statistical relevance of values at 1%, 5%, and 10% levels in that order.

### Empirical tests

A set of empirical tests is performed ahead of the principal empirical analysis. Considering this is a panel investigation, cross-sectional dependency tests are employed through the approaches of^50^ and^51,52^. The results for the approaches, as displayed in Table 3, show statistical evidence of cross-sectional dependence since the null hypothesis is rejected in all cases. Thus, this result paved the way for applying the right stationarity and cointegration tests.

**Table 3:** Cross-sectional Dependence Test.

Methods	Breusch and Pagan^50^ LM Test	Pesaran^51^ CD Test	Pesaran^52^ LM Test
Model (1)	234.80***	15.14***	32.99***
*P*-value	(0.000)	(0.000)	(0.000)
Model (2)	235.67***	15.18***	33.12***
*P*-value	(0.000)	(0.000)	(0.000)
Model (3)	95.79***	− 0.627	11.54***
*P*-value	(0.000)	(0.531)	(0.000)
Model (4)	99.30***	0.865	12.08***
*P*-value	(0.000)	(0.387)	(0.000)

Computed by the author. ***, **, and * reflect the statistical relevance of values at 1%, 5%, and 10% levels in that order.

Because of the cross-sectional evidence, the unit root test is first tested using the^53^ Im et al. (2003) technique and then followed by a more compatible stationary technique by^51^ Pesaran (2007). The former method shows that all variables are only integrated at the order of one, while the result of the latter approach shows some evidence of mixed stationarity, i.e. both at the level and after the first difference. However, the overwhelming conclusive evidence supports the stationarity at I (1) for all the variables (see Table 4). Moreover, the cointegration of the examined models is investigated by applying the error correction type of^54^ Westerlund (2007), and the result is indicated in Table 5.

**Table 4:** Unit root results.

Variables list	Pesaran^51^		Im et al.^53^	
	Trend & intercept model		Trend & intercept model	
	I(0)	I(1)	I(0)	I(1)
LnCM	− 1.894	− 3.580***	− 1.942	− 3.734***
LnGDP	− 2.939*	− 4.142***	− 1.104	− 4.125***
LnGFL	− 2.549	− 3.840***	− 2.524	− 4.063***
LnFEX	− 3.504**	− 4.840***	− 2.172	− 2.850**
LnUB	− 1.812	− 2.949**	0.414	4.200***
LnGQ	− 2.986**	− 4.351***	− 2.005	− 4.052***
LnGQGFL	− 2.682	− 4.870***	− 1.874	− 3.947***

Computed by the author. ***, **, and * reflect the statistical relevance of values at 1%, 5%, and 10% levels in that order.

**Table 5:** Cointegration Test Westerlund^54^.

Models	Group stat		Panel stat	
	Gτ	Gα	Pτ	Pα
Model (1) Stat	− 1.552***	− 2.211***	− 5.380***	− 2.751***
Robust *p*-value	(0.000)	(0.000)	(0.000)	(0.000)
Model (2) Stat	− 1.280***	− 2.260***	− 4.885***	− 3.015***
Robust *p*-value	(0.000)	(0.000)	(0.000)	(0.000)
Model (3) Stat	− 1.965***	− 0.473	− 1.528***	− 0.300***
Robust *p*-value	(0.000)	(1.000)	(0.000)	(0.000)
Model (4) Stat	− 2.110***	− 0.490***	− 1.822***	− 0.286***
Robust *p*-value	(0.000)	(0.000)	(0.000)	(0.000)

Computed by the author. ***, **, and * reflect the statistical relevance of values at 1%, 5%, and 10% levels in that order.

#### Empirical analysis

In this study, the main empirical analysis employed is the estimator of AMG (augmented mean group), which was put forward in the studies of^55,56^. Although the common correlated-effect approach, like the AMG, takes account of the cross-sectional effect, the latter is more efficient in considering the effect of the unobserved common factors, especially in estimating the cointegrating nexus for the country-specific case. Three steps are involved in the layout of this empirical approach, as detailed in the aforementioned studies^55,56^. Meanwhile, since this study aims to examine the economic and environmental aspects of natural gas flaring alongside other direct and indirect factors, two empirical models are utilised accordingly under each of the economic and environmental parts.

The economic models:

Model 1 $${\text{GDP }} = f\left( {{\text{GFL}},{\text{ FEX}},{\text{ UB}},{\text{ GQ}},\mu } \right)$$

Model 2 $${\text{GDP }} = f\left( {{\text{GFL}},{\text{ FEX}},{\text{ UB}},{\text{ GQGFL}},\mu } \right)$$

The environmental models:

Model 3 $${\text{CM }} = f\left( {{\text{GDP}},{\text{ GFL}},{\text{ FEX}},{\text{ UB}},{\text{ GQ}},\mu } \right)$$

Model 4 $${\text{CM }} = f\left( {{\text{GDP}},{\text{ GFL}},{\text{ FEX}},{\text{ UB}},{\text{ GQGFL}},\mu } \right)$$

where GFL *GQ = GQGFL (as previously mentioned), GQ is a function of regulatory policy and government effectiveness, and *µ* is the normally distributed error term mean = 0 and constant variance = σ^2^. The long-run panel estimation of the models 1–4 is produced via the AMG estimator for seven (7) cross sections (i.e. Russia, Iraq, Iran, the United States, Algeria, Venezuela, and Nigeria) over the annual period of nineteen (19) years (i.e. 2002–2020).

Also, models 1–4 are similarly employed to produce results for each country under investigation. This is along with applying the panel Granger causality approach by^57^ to provide robust evidence.

## Discussion of findings

This section is dedicated to the discussion of the results (economic and environmental effects) for the panel and country-wise.

### Panel result: economic aspect

Following the result of the long-run panel estimation via the AMG estimator, as illustrated in Table 6, there are economic and environmental dimensions of the investigation. First, from the economic perspective, there is the statistical revelation that fuel energy export positively impacts economic growth by elasticity of ~ 0.22 (in model 1) and ~ 0.24 (in model 2). This implies a less disproportionate increase in economic growth in response to the increase in fuel energy export, especially in the long run. The positive response between the two variables is likely associated with the channelling of earnings from energy to economic components such as consumption and investment in goods and services. Considering that the countries under investigation are major oil-producing states, the contribution of the revenue from oil production and export to economic growth is not unexpected, as evident in the existing literature^58,59^. Moreover, the result reveals a tendency for a negative impact of natural gas flaring on economic output but is not statistically significant as the urban population increase and government quality does not also significantly affect economic prosperity in the long run.

**Table 6:** Long-run estimations.

Variables	Long-run estimations (AMG)			
	Economic impacts (GDP Dependent variable)		Environmental impacts (CO_2_ Dependent variable)	
	Model 1	Model 2 (Interactions)	Model 3	Model 4 (Interactions)
LnGDP	–	–	0.090 (0.000) ***	0.072 (0.001) ***
LnGFL	− 0.047 (0.589)	− 0.073 (0.302)	0.069 (0.097) *	0.075 (0.010) ***
LnFEX	0.218 (0.016) **	0.243 (0.038) **	− 0.032 (0.341)	0.021 (0.000) ***
LnUB	0.479 (0.624)	0.389 (0.682)	1.520 (0.000) ***	1.725 (0.000) ***
LnGQ	0.042 (0.771)	–	− 0.072 (0.387)	–
LnGQGFL	–	− 0.010 (0.898)	–	− 0.065 (0.081) *
Constant	1.111 (0.000) ***	1.129 (0.000) ***	− 16.505 (0.000) ***	− 21.439 (0.007) ***
Observations	119	119	119	119
No of Groups	7	7	7	7

*P*-values for coefficients are in brackets.

***, **, and * reflect the statistical relevance of values at 1%, 5%, and 10% levels in that order.

#### Panel result: environmental aspect

The environmental perspective, as indicated in the last two columns of Table 6, offers an interesting dimension. The result shows that economic growth, natural gas flaring, fuel energy export, and urbanisation all positively and statistically significant impacts on carbon emission trends in the panel examination. With the exemption of urbanisation (elastic in relationship), there is an inelastic relationship between the abovementioned variables and carbon emission. For the economic growth and environmental degradation nexus, all the examined countries with the exemption of the United States of America are developing economies, thus signalling that the economic growth in these countries is yet to be decoupled from carbon emissions. Considering that the countries are mostly members of the Organization of the Petroleum Exporting Countries (OPEC), several studies have revealed that economic growth spur environmental degradation^60,61^. Although similar studies exist in the literature that examines the environmental effect of urbanisation, fuel energy export, and natural gas flaring, the results are generally inconclusive. For instance^61^, failed to establish a significant relationship between OPEC members' oil and gas export and carbon emissions. Desirably, the result further showed that applying government quality in tackling the problem of natural gas flaring reduces carbon emissions in the long run. Specifically, a 1 percent scaling up in government quality to tackle natural gas flaring can reduce carbon emissions by ~ 0.07 percent.

### Country-specific result: economic and environmental aspects

The country-wise results of the economic (models 1 and 2) and environmental (models 3 and 4) aspects are implied in Table 7.

**Table 7:** Country-specific estimations.

Variables	Russia	Iraq	Iran	USA	Algeria	Venezuela	Nigeria
Model 1 (Economic impacts)							
LnGDP	–	–	–	–	–	–	–
LnGFL	− 0.043	− 0.285	0.364**	0.105**	− 0.039	− 0.105	− 0.237
LnFEX	0.301	0.296	0.053	− 0.088*	4.491*	0.497	0.237
LnUB	− 10.923**	1.845**	− 3.721***	2.637***	0.349	1.194	− 0.459
LnGQ	− 0.063**	0.191	− 0.560***	0.490***	− 0.027	0.314	− 0.141
LnGQGFL	–	–	–	–	–	–	–
	Model 2 (Economic impacts)						
LnGDP	–	–	–	–	–	–	–
LnGFL	− 0.064	− 0.219	0.338*	0.037	− 0.027	− 0.135	− 0.233
LnFEX	0.295	0.532	0.003	− 0.106**	4.703**	0.507	0.215
LnUB	− 10.642**	1.861***	− 3.752***	2.457***	0.320	0.926	− 0.420
LnGQ	–	–	–	–	–	–	–
LnGQGFL	− 0.021**	− 0.266	− 0.205***	0.245***	0.043	0.181	− 0.045
	Model 3 (Environmental impacts)						
LnGDP	0.090***	0.079	0.061***	0.850***	0.095**	0.125**	− 0.203***
LnGFL	0.000	0.128	0.037	0.003	0.135***	0.031	0.201**
LnFEX	− 0.104	− 0.112	0.002	0.026	− 0.876	0.026	0.727**
LnUB	1.105	1.356*	1.041***	− 4.368***	1.360***	2.306**	2.806***
LnGQ	− 0.007	0.144	0.134***	− 0.054	− 0.050	− 0.372**	− 0.329*
LnGQGFL	–	–	–	–	–	–	–
	Model 4 (Environmental impacts)						
LnGDP	0.090***	− 0.000	0.060***	0.856***	0.097***	0.099**	− 0.211***
LnGFL	− 0.002	0.120	0.045	0.012	0.109***	0.060	0.198**
LnFEX	− 0.105	0.016	0.016	0.027	− 0.981	0.023	0.686**
LnUB	1.114	1.579**	1.066***	− 4.398***	1.520***	2.465***	2.854***
LnGQ	–	–	–	–	–	–	–
LnGQGFL	− 0.002	− 0.090	0.048***	− 0.0291	− 0.075	− 0.215***	− 0.123*

Computed by the author. ***, **, and * reflect the statistical relevance of values at 1%, 5%, and 10% levels in that order.

In the case of Russia, economic growth is inhibited by the government's quality process in natural gas flaring, urbanisation, and government quality at a 5% statistically significant level. Nevertheless, statistical evidence supports that improvement of government quality promotes economic prosperity. The evidence, especially for the moderating effect of government quality in natural gas flaring, could be explained by the economic cost of transitioning from the business-as-usual approach to natural gas production. Considering that retrofitting of existing natural gas plants, the introduction of relevant technological innovation in the production mechanism, among other reasons, could be cost-intensive, especially in the short term, thus possibly creating more economic burden. Furthermore, the rural–urban movement trend could also undermine the contribution of economic opportunities and potential concentrated in the country's rural areas, thus causing a significant economic downturn inferred by the result. Moreover, GDP is the only significant contributor to environmental degradation, with its impact signalling a decline in environmental quality as economic growth increases.

The result implies that urbanisation plays critical economic and environmental roles in Iraq. Specifically, the result revealed that urbanisation spurs economic prosperity in the country while worsening environmental quality. Iraq is a major oil-producing, and oil-dependent country with enormous revenue from fossil fuel energy, major development across the country is mainly concentrated in the urban area, which in turn constitutes or attracts significant economic activities. Generally, urbanisation's economic and environmental impact has so far generated an inconclusive outcome in the literature^62–64^. Consequently, the associated adverse environmental effect is not unexpected.

For Iran, natural gas flaring is seen as a positive contributor to economic growth, while urbanisation, government quality, and the interaction of government quality and natural gas flaring hinder economic growth. These observations, especially for the effect of government quality and its interaction with natural gas flaring, are not surprising given the characteristics of the system of government in the country, entrenched in Islamic and traditional beliefs. So, changing the orthodoxically bureaucratic processes, including that involved in natural gas flaring production, could also constitute havoc on the economy. Similarly, this is also the reason the statistical evidence further revealed that economic growth, urbanisation, and the interaction between government quality and natural gas flaring hampers environmental quality in the country. The statistics suggest that the Islamic Republic of Iran has continued to record low performance in government effectiveness and regulatory quality^65^.

The result of the United States is also not far from expectation. Natural gas flaring, urbanisation, and government quality are positive contributors to economic growth, while fuel energy export is detrimental to the economy. The negative impact of fuel energy export might be related to the fact that the country has always been a net energy importer until 2020 when energy imports declined (due to the coronavirus pandemic) by 13%^66^. As for the desired impact of government quality on economic growth, that could better be explained by the level of advancement in government effectiveness and regulatory quality as reported by the^65^. Furthermore, urbanisation is a significant promoter of environmental quality, while economic growth worsens environmental quality. Although several studies have revealed the positive effect of economic growth on environmental quality or the validity of the Environmental Kuznets curve for the United States^67,68^, this study is, however, short of such an endeavour.

As per Algeria and Venezuela, fuel energy export promotes economic growth while causing environmental deterioration in Algeria. Moreover, economic growth and urbanisation contribute to environmental degradation in Algeria and Venezuela. Meanwhile, as an unexpected observation, government quality moderates natural gas flaring to cause improvement in environmental quality in Venezuela.

Lastly, similar to the case of Venezuela, there is no significant economic impact from natural gas flaring, fuel energy export, urbanisation, and government quality in Nigeria. However, statistically significant evidence shows that natural gas flaring, fuel energy export, and urbanisation worsen environmental quality in Nigeria, while economic growth, government quality, and its moderating effect all promote environmental quality in the country. Therefore, the undesirable environmental effects of energy-related factors could be associated with the perception of the resource curse hypothesis, which is peculiar to resource-rich countries such as Nigeria^69^.

### Evidence of granger causality

In Table 8, evidence of Granger causality is implied. As illustrated, statistical evidence supports that historical information on GDP, natural gas flaring, urbanisation, government quality, and the interaction of government quality and natural gas flaring all Granger cause carbon emission in the estimated panel countries. Meanwhile, there is a bidirectional Granger causality from carbon emission to natural gas flaring, urbanisation, and natural gas flaring, while a one-way direction from GDP to natural gas flaring, fuel energy export, and urbanisation. Moreover, there are other Granger causality relationships among the explanatory variables.

**Table 8:** Panel causality evidence.

Variables	W-stat							Causality flow
	LnCE	LnGDP	LnGFL	LnFEX	LnUB	LnGQ	LnGQGFL	
LnCM	_	0.947	4.679***	3.3559**	61.935***	0.971	0.440	$LnCO2\to LnGFL,LnFEX,LnUB$
LnGDP	3.589***	_	4.125***	2.809**	49.004***	2.077	0.925	$LnGDP\to LnCO2,LnGFL,LnFEX,LnUB$
LnGFL	3.713***	1.085	_	3.736***	7.436***	0.952	1.010	$LnGFL\to LnCO2,LnFEX,LnUB$
LnFEX	1.942	0.541	2.327	_	6.916***	2.896	3.187	$LnFEX\to LnUB$
LnUB	3.363***	1.173	4.372***	3.922***	_	1.750	1.199	$LnUB\to LnCO2,LnGFL, LnFEX$
LnGQ	5.232**	2.315	5.608***	5.205**	9.192***	_	2.635	$LnGQ\to LnCO2, LnGFL, LnFEX, LnUB$
LnGQGFL	7.690***	1.891	5.988***	4.115	8.667***	2.637	_	$LnGQGFL\to LnCO2,LnGFL,LnUB$

Computed by the author. ***, **, and * reflect the statistical relevance of values at 1%, 5%, and 10% levels in that order.

## Conclusion and policy measure

Considering that there is limited literature about the economic and environmental effects of natural gas flaring and government quality, among other factors, the current study looked at the case of the world's largest natural gas flaring countries. Therefore, the investigation was performed for a dataset covering 2002–2020 for the case of the world's seven countries with the highest volume of natural gas flaring (i.e. Russia, Iraq, Iran, the United States, Algeria, Venezuela, and Nigeria). While a series of preliminary tests are found supportive, the estimator of AMG technique^55,56^, alongside the Dumitrescu and Hurlin^57^ approach, provided detailed empirical results.

From the economic perspective, the result revealed that fuel energy export positively impacts economic growth by ranging from an elasticity of ~ 0.22 to ~ 0.24. The result further revealed that environmental quality in the panel is hampered by increased economic growth, natural gas flaring, fuel energy export, and urbanisation. However, only urbanisation produced an elastic relationship, while an inelastic relationship ensued between the variables and carbon emission.

For the country-wise inference, economic growth positively impacted urbanisation (only in Iraq and the USA), natural gas flaring (only in Iran and the USA), government quality (only in the USA), and fuel energy export (only in Algeria). In contrast, declining economic growth is ensured by increasing urbanisation in Russia and the USA, increasing fuel energy export in the USA, and increasing government quality in Russia. Meanwhile, environmental quality is worsened through intense carbon emission from increased urbanisation activity (in Iraq, Iran, Algeria, and Nigeria), increased fuel energy export (in Nigeria), increased natural gas flaring (in Algeria and Nigeria), increased GDP (in Russia, Iran, USA, Algeria, and Venezuela), and high government quality (in Iran). However, an increase in GDP (Nigeria), an increase in urbanisation (in the USA), and an increase in natural gas flaring (in Algeria and Nigeria) dampen environmental quality. Moreover, the Granger causality result revealed bidirectional causality from carbon emission to natural gas flaring and urbanisation.

### Related policy

Considering that all the examined countries with the exemption of the United States of America are developing economies, policy toward escalating sustainable development through decoupling economic growth from carbon emission should be an utmost priority for decision-makers. Sustainable growth could be achieved through sector-wide adoption of green socioeconomic practices and cultural/firm-level green behavioural approaches. In addition, a policy-specific approach toward reducing natural gas flaring and improving government quality in the examined countries is not less desirable. Specifically, a gas flaring price targeting natural gas companies should be more effective in mitigating gas flaring than the wider ‘carbon price’ or pollution price/tax policy. Moreover, deficiency in the quality of government (responsible for poor economic and environmental performances) in the examined countries could be improved through a continuous policy review that advances institutional effectiveness, reduce bureaucratic drawbacks, and increase the digitisation of sector-wide economic activities, among others.

## Research limitations and future direction

Although the current study has contributed to the growing literature on the developments surrounding gas flaring from both environmental and economic perspectives, the study still has some limitations mainly from the aspect of the current scope. The leading countries that have been analysed in this study jointly account for about 65% of the total global gas flaring, though a good proportion of the entire problem to begin research with, but that does not in any wise suggest that the remaining flaring activities in other countries should be side-lined. As such, there are still opportunities for future studies to broaden the current scope, perhaps, using other analytical approaches/methodologies to widen the suggested policy frameworks against gas flaring towards reaching a more sustainable environment.

## Data Availability

Data is avialable upon request and in online database. Please contact andrew.alola@hotmail.com.

## Abbreviations

ADMS: Atmospheric dispersion modelling system
AMG: Augmented mean group
ARDL: Auto-regressive distributed lags model
BH: Bayer–Hanck cointegration test
CD: Cross-sectional dependence
CO_2_: Carbon dioxide
CH_4_: Methane
CM: Carbon emission
DALYs: Disability-adjusted life years
ECM: Error correction model
EHA: Evenemential historical analysis
FEX: Fuel export
GC: Granger causality tests
GFL: Gas flaring
GDP: Gross domestic product
GHG: Greenhouse gas
GQ: Government quality
GQGFL: Interaction of government quality and gas flaring
GSE: Gross specific enthalpy
GTL: Gas to liquid
LM: Lagrange multiplier
LNG: Liquefied natural gas
OPEC: Organization of the Petroleum Exporting Countries
RA: Reference approach
UB: Urbanisation
WHO: World Health Organization
ZRF: Zero routine flaring
